# Discovery of Artemisinin (Qinghaosu)

**DOI:** 10.3390/molecules14125362

**Published:** 2009-12-21

**Authors:** Fulong Liao

**Affiliations:** Institute of Chinese Materia Medica, China Academy of Chinese Medical Sciences, Beijing, 100700, China; E-Mail: liaofulong100@yahoo.com.cn

Artemisinin (Qinghaosu), a new antimalarial drug, was discovered in China in the early 1970s. The discovery of artemisinin is attributed to You-You Tu, at that time a middle-aged phytochemist working in the Institute of Chinese Materia Medica, China Academy of Traditional Chinese Medicine. The following paragraph provides a brief summary of her discovery.

In the late 1960s and 1970s, Tu was the head of an antimalarial research group, which comprised both phytochemical and pharmacological researchers. She led her group of young scholars in the extraction and isolation of constituents with possible antimalarial activities from Chinese herbs. During the first stage of this research, her group investigated more than 2,000 Chinese herb preparations and identified 640 recipes that might have some antimalarial activities. More than 380 extracts, obtained from some 200 Chinese herbs, and including extracts from *Artemisia annua* L., were tested against a rodent malaria model. However, progress was not smooth and no significant results were obtained at first. The turning point came when an *Artemisia* extract showed a promising degree of inhibition against parasite growth, consistent with activity which had been reported for this species in “A Handbook of Prescriptions for Emergencies” by Ge Hong (Jin Dynasty, 284-346 AD). Tu brilliantly modified the extraction technique to perform it at low temperature, rather than using heating, as was conventional. Much better antimalarial activity was obtained after switching to the lower temperature procedure, and she found that the most effective preparation came from the leaves of *Artemisia annua* L., as evidenced by its significant inhibition of mouse malaria *P. berghei*. Unfortunately, this extract also appeared to be toxic to the animals, despite its effectiveness against malaria. However, Tu was able to separate the extract into an acidic portion, which contained no antimalarial activity, and a neutral extract, which exhibited both reduced toxicity and improved antimalarial activity. During the Cultural Revolution, there were no facilities for performing trials of new drugs, so, in order to help malaria sufferers, Tu and her colleagues bravely acted as the first group of volunteers and took the new extract themselves. After their first human experiments, Tu and her team went to Hainan to verify the efficacy of the extract clinically, and carried out antimalarial trails with patients infected with both *P. vivax* and *P. falciparum*. These clinical trials produced encouraging feedback, achieving a rapid disappearance of fever and parasites from the blood as compared with the control group using chloroquine. Tu next investigated the isolation and purification of the active component from *Artemisia annua* L. Eventually, in 1972, her team identified a colourless crystalline substance with a molecular weight of 282 Da, a molecular formula of C_15_H_22_O_5_ and a melting point of 156-157 °C, as the active principal and named it “Qinghaosu” (“Qinghao” is the Chinese name of *Artemisia annua* L., and “su” means basic element). The stereochemistry and structure of Qinghaosu, was later determined by Tu in 1975 with the assistance of the Institute of Biophysics, Chinese Academy of Sciences, as that of a sesquiterpene lactone. The structure was first published in 1977 [2], and both the new molecule and the paper were quickly cited by Chemical Abstracts in the same year (C.A. **1977**, *87*, 98788g). However, because of the prevailing environment, not many papers concerning Qinghaosu were published and these were mostly in Chinese. In addition, authors were not always identified individually in some of the early papers [1,2,3,4,5,6,7,8,9,10,11,12,13,14,15,16,17,18,19,20,21,22,23,24,25,26,27,28,29,30,31,32,33,34,35], which is perhaps why the name of You-You Tu is not as well known internationally as that of her discovery, Qinghaosu. 

*Plasmodium falciparum* has been a life-threatening disease for thousands of years and still threatens millions of lives every year in many parts of the World, particularly in Africa. After a failed international attempt to eradicate malaria in the 1950s, the disease rebounded, largely due to the emergence of parasites which were resistant to the existing antimalarial drugs of the time, such as chloroquine. Artemisinin was a new antimalarial agent with a totally different chemical structure and a higher efficacy, as compared with the conventional drugs against which resistance has been acquired and the successful application of Qinghaosu (artemisinin) and its derivatives for treating several thousand malaria patients in China attracted worldwide attention in the 1980s [36]. The discovery of artemisinin has since been recognized as a significant milestone in the human journey towards conquering malaria. In 2005, WHO announced a switch in strategy to artemisinin combination therapy (ACT). ACT is currently widely used, saving many lives, mostly of children in Africa (the remedy impressively reduces the intensity of malaria in Africa due to its anti-gametocyte activity). None of this could have been achieved without the initial discovery of Qinghaosu.

Qinghaosu was awarded the status of “national scientific discovery” by the Committee of Science and Technology of China in 1979. During a special program for research and training in tropical diseases of the fourth meeting of the scientific working group on the chemotherapy of malaria (sponsored by UNDP/World Bank/WHO) in Beijing in 1981, Prof. Tu was invited to deliver a lecture on Qinghaosu. Chinese new drug certificates were issued both for Qinghaosu and for its derivatives which had been developed by Prof. Tu and her team [36,37,38,39,40]. Since the 1970s, Qinghaosu and Prof. You-You Tu have won more than ten awards at a national level in China [43,44,45,46,47,48,49,50,51,52,53,54,55]. Prof. Tu is now the director of the Qinghaosu Research Center, Institute of Chinese Materia Medica, China Academy of Chinese Medical Sciences (the renamed China Academy of Traditional Chinese Medicine), and a life-long professor of the academy. On the occasion of Prof. You-You Tu’s 80th Anniversary, we would like to express our cordial respect to the discoverer of artemisinin (Qinghaosu), the great master playing with molecular diversity, for her outstanding contribution to the mankind. 
